# Aberrant methylation limits antitumoral inflammation in lung adenocarcinoma by restricting RIPK3 expression

**DOI:** 10.1126/sciadv.adz9227

**Published:** 2026-01-21

**Authors:** Deepti Agrawal, Katarina Cisarova, Sebastian Vosberg, Fabian Allmendinger, Enkhtsetseg Munkhbaatar, Nadia Dandachi, Francisco Jose Fernandez Hernandez, Marta Tonietto, Vanessa Jäger, Martina Anton, Eva C. Keller, Moritz Jesinghaus, Anna-Lena Meinhardt, Verena Haefner, Tobias Stoeger, Katja Steiger, Nicholas McGranahan, Michael A. Dengler, Adam Wahida, Philipp J. Jost

**Affiliations:** ^1^ Medical Department III, School of Medicine, Technical University of Munich, Munich, Germany.; ^2^ Division of Oncology, Medical University of Graz, Graz, Austria.; ^3^ Department of Medicine III, University Hospital, Ludwig-Maximilians-Universität München, Munich, Germany.; ^4^ German Cancer Consortium, Partner site Munich, Munich, Germany.; ^5^ German Cancer Research Center, Heidelberg, Germany.; ^6^ Department of Radiation Oncology, Heidelberg University Hospital, Heidelberg University, Heidelberg, Germany.; ^7^ Heidelberg Institute for Radiation Oncology (HIRO) and National Center for Radiation Research in Oncology (NCRO), Heidelberg, Germany.; ^8^ National Center for Tumor Diseases (NCT), NCT Heidelberg, a Partnership Between DKFZ and Heidelberg University Hospital, Heidelberg, Germany.; ^9^ Heidelberg Ion-Beam Therapy Center (HIT), Department of Radiation Oncology, Heidelberg University Hospital, Heidelberg University, Heidelberg, Germany.; ^10^ Department of Surgery, TUM School of Medicine, Technical University Munich, Munich, Germany.; ^11^ Institute for Experimental Haematology, TUM School of Medicine and Health, Technical University Munich, Munich, Germany.; ^12^ Institute of Molecular Immunology, School of Medicine, Technical University of Munich, University Hospital rechts der Isar, Munich, Germany.; ^13^ Institute of Pathology, Technical University of Munich, Munich, Germany.; ^14^ Institute of Pathology, University Hospital Marburg, Marburg, Germany.; ^15^ Institute of Lung Health and Immunity (LHI), Comprehensive Pneumology Center, Helmholtz Munich, Member of the German Center for Lung Research, Munich, Germany.; ^16^ German Cancer Consortium (DKTK), German Cancer Research Center (DKFZ), Heidelberg, Germany.; ^17^ Cancer Research UK Lung Cancer Center of Excellence, University College London Cancer Institute, Paul O’Gorman Building, London WC1E 6BT, UK.; ^18^ Cancer Genome Evolution Research Group, University College London Cancer Institute, University College London, London, UK.; ^19^ Institute of Metabolism and Cell Death, Helmholtz Munich, Neuherberg, Germany.; ^20^ Universitäres Comprehensive Cancer Center (Krebszentrum) Graz, LKH-Univ. Klinikum Graz, Graz, Austria.; ^21^ BioTechMed-Graz, Graz, Austria.

## Abstract

Evasion of programmed cell death is a critical hallmark of cancer. However, the contribution of inflammatory forms of cell death in lung carcinogenesis and their effects on the composition of the tumor-immune microenvironment remain unclear. Our multi-omics analyses of samples from patients with primary lung adenocarcinoma revealed that necrosome signaling is repressed because of reduced expression of receptor-interacting protein kinase 3 (*RIPK3*). Distinct methylation signatures, both in the *RIPK3* promoter and nonpromoter regions, correlated with lower transcription levels of *RIPK3*. This resulted in limited expression of inflammatory genes, advanced histologic features, reduced immune cell invasion, and decreased patient survival. Mechanistically, we confirmed the tumor-suppressive role of necrosome signaling through the genetic deletion of *Ripk3* in two independent, clinically relevant mouse models of lung adenocarcinoma. Functionally, RIPK3 shaped a diverse immune environment by promoting the invasion of innate and adaptive immune cells in patient samples and experimental mice. Thus, RIPK3-mediated inflammatory signaling enhances a diverse immune microenvironment and hinders progression in lung adenocarcinoma.

## Teaser

Aberrant methylation silences RIPK3 in lung adenocarcinoma, limiting necroinflammation and immune defense against tumors.

## INTRODUCTION

Lung cancer presents with (patho-)histological and genomic diversity, which determines its distinct molecular and clinical subtypes (*1*). Current treatment approaches use known cancer driver alterations and immune checkpoint inhibitors to inform individualized therapy (*2*). In spite of these efforts, lung cancer remains the leading cause of cancer-related fatalities globally (*3*). Cancer cells frequently develop strategies to avoid cell death pathways, most importantly apoptosis (*4*). While the role of apoptosis in lung cancer has been studied (*5*), the consequences of other regulated cell death forms and their influence on inflammatory processes in lung cancer remain less well understood (*6*).

Necroptosis is engaged downstream of a series of cell surface receptors, including tumor necrosis factor receptor (TNFR) family members like TNFR1, tumor necrosis factor ligand superfamily member 10 (TRAIL) receptor, death receptor 6, tumor necrosis factor receptor superfamily member 6 (FAS), as well as Toll-like receptor 3 (TLR3) and TLR4, or through intracellular pattern recognition receptors such as the Z-DNA binding protein (*6*–*9*). Receptor-interacting protein kinase 1 (RIPK1) and RIPK3 initiate necroptosis by recruiting the mixed lineage kinase domain–like (MLKL) pseudokinase, which ultimately executes cell death (*10*). Notably, several studies have linked necroptosis to inflammatory conditions, including viral infections (*11*, *12*), sepsis (*13*), and inflammatory bowel disease (*14*–*17*). Beyond its canonical role in necroptosis, RIPK3 has been increasingly recognized for also mediating non–cell-death functions, including the regulation of transcriptional and inflammatory signaling (*18*, *19*). In cancer, RIPK3 has a bifurcated function across cancer subtypes, which appears to be strongly context dependent (*20*). While RIPK3 acts as a tumor suppressor in acute myeloid leukemia (AML) (*21*), it acts to promote pancreatic cancer (*22*) and liver cancer (*23*). This suggests that tissue-specific cues govern the effects of RIPK3-dependent signaling in malignant disease (*22*).

To investigate RIPK3’s role in lung adenocarcinoma (LUAD), the most prevalent lung cancer subtype (*24*), we took advantage of molecular profiles from multiregion exome, transcriptome, and DNA methylation data from The TRAcking Non-small Cell Lung Cancer Evolution Through Therapy (TRACERx) consortium (*25*), and The Cancer Genome Atlas (TCGA) Research Network (*26*, *27*). Coupled with functional data from lung cancer mouse models, we establish that RIPK3 acts as a tumor suppressor in LUAD.

## RESULTS

### RIPK3 expression is progressively lost in human lung cancer

**Fig. 1.**
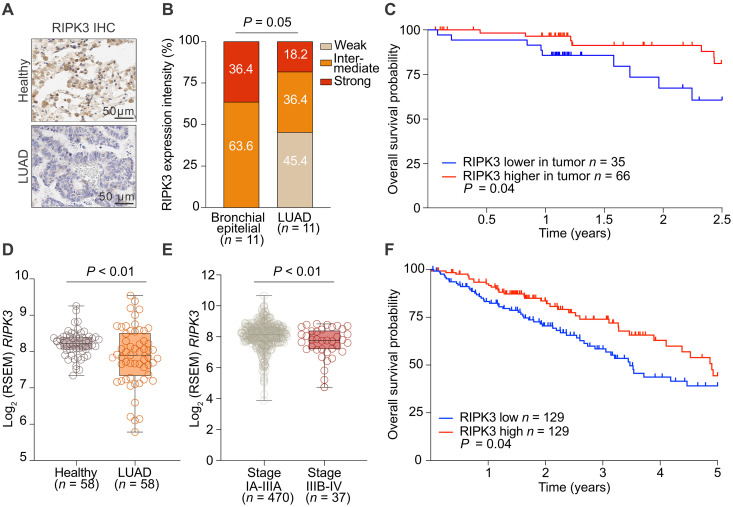
RIPK3 is progressively lost in human lung cancer. (**A**) Representative images of immunohistochemistry (IHC) RIPK3 staining in LUAD (bottom) and paired healthy tissue (top). (**B**) Quantification of RIPK3 staining across LUAD samples and matched healthy adjacent tissue, classified as weak, intermediate, or strong. (**C**) Kaplan-Meier plot showing the differences in overall survival of CPTAC-LUAD cases with reduced expression of RIPK3 protein in tumor compared to that in matched normal sample versus cases with increased expression of RIPK3 protein in tumor compared to that in matched normal tissue. (**D**) *RIPK3* mRNA levels in TCGA-LUAD and paired healthy tissue. (**E**) *RIPK3* mRNA levels in TCGA-LUAD early- versus late-stage tumor samples. (**F**) Kaplan-Meier plot showing the differences in overall survival of TCGA-LUAD cases with RIPK3^hi^ versus RIPK3^lo^ expression, as defined by upper and lower quartiles. Fisher’s exact test was applied on the number of samples in each specific category (B), Firth’s penalized Cox proportional hazards model was applied to data in (C), data in (D) were analyzed by the Wilcoxon signed-rank test (paired), data in (E) were analyzed by the Mann-Whitney rank sum test, and data in (F) analyzed by the log-rank test.

We explored how regulated necrosis and its related inflammation affect lung cancer, specifically examining the function of the key checkpoint within the necrosome protein complex, RIPK3. Initially, we analyzed RIPK3 protein expression, a key regulator of the necrosome, in 11 primary LUAD samples compared to that in adjacent healthy tissue (Fig. 1, A and B). We found that RIPK3 expression was significantly reduced in LUAD cells (*P* = 0.05, Fisher’s exact test) (Fig. 1, A and B). We further validated these findings using RIPK3 protein expression in the LUAD Clinical Proteomic Tumor Analysis Consortium (CPTAC) cohort (*n* = 101 matched tumor-normal pairs), where 35% of patients showed lower RIPK3 expression in the tumor compared to that in the matched normal tissue. These patients had significantly poorer overall survival [hazard ratio (*HR*) = 2.78; 95% confidence interval (CI), 1.06 to 7.57; *P* = 0.04, Firth’s penalized Cox model] at 2.5 years of follow-up (Fig. 1C). Last, a meta-analysis with an independent cohort from Lehtiö *et al.* (*28*) (*n* = 142) confirmed that low tumor RIPK3 expression consistently associated with worse survival (*HR *= 2.14; 95% CI, 1.02 to 4.58; *P* = 0.04; fig. S1A). We then used gene expression datasets collected within TCGA for patients with LUAD (*n* = 515) and paired tumor/control samples (*n* = 58). We detected significantly lower *RIPK3* mRNA expression levels compared to those in matched healthy tissue (tumor samples, mean = 7.90; and normal samples, mean = 8.22; *P* < 0.01, Mann-Whitney rank sum test) (Fig. 1D). Patients with clinically advanced stage tumors showed lower *RIPK3* expression (early-stage samples, mean = 8.15; and late-stage samples, mean = 7.75; *P* = 0.01, Mann-Whitney rank sum test) (Fig. 1E and fig. S1B). Furthermore, when stratifying patients according to *RIPK3* expression (RIPK3^lo^ = 129 and RIPK3^hi^ = 129, stratified by quartiles of gene expression), we found a significantly inferior overall survival for RIPK3^lo^ patients (χ^2^ = 3.56; df = 1, *P* = 0.04, log-rank test) (Fig. 1F and fig. S1C), a finding recently reported for squamous cell lung cancer (*29*).

### Aberrant methylation patterns result in repression of *RIPK3* expression in LUAD

To investigate how *RIPK3* is repressed in patients with LUAD, we analyzed genomic alterations (data from *n* = 513) and copy number changes (data from *n* = 526) of *RIPK3* within the TCGA cohort. We identified nonsynonymous *RIPK3* mutations in only 3 of the 513 patients (0.6%), which did not significantly affect *RIPK3* mRNA expression (*P* = 0.40, Mann-Whitney rank sum test). Additionally, deep deletions and high-level amplifications were observed in 14 of the 526 cases (2.7%; 13 high level gains, one deep deletion), yet these also had no significant effect on *RIPK3* mRNA expression (high level gain, mean = 339.84; deep deletions, mean = 84.70; no copy number variations (CNV), mean = 304.36; deep deletions versus no CNVs, *P* = 0.12; high level gains versus no CNVs, *P* = 0.58, Mann-Whitney rank sum test) (table S1). Given that genetic alterations could not account for the notable decrease in *RIPK3* expression throughout LUAD progression, we focused on epigenetic factors, specifically DNA methylation of CpG sites within or upstream of the *RIPK3* gene locus, as a possible mechanism for its regulation.

**Fig. 2.**
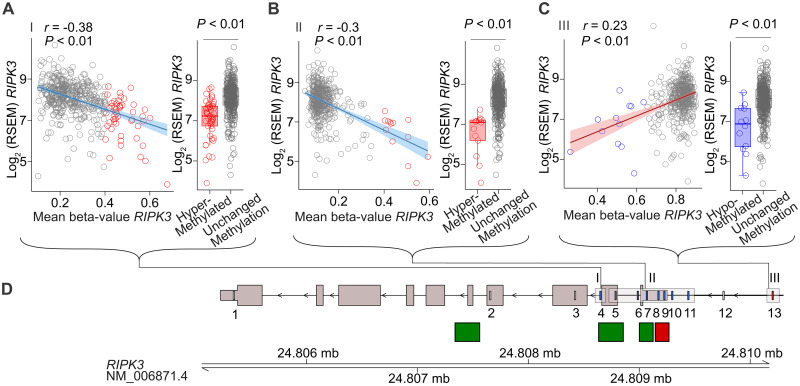
DNA methylation and *RIPK3* mRNA expression patterns in LUAD and healthy tissue. (**A**) Cluster I was hypermethylated in 49 LUAD samples and associated with significantly reduced *RIPK3* expression. (**B**) Cluster II showed hypermethylation in 13 LUAD cases, with most samples displaying down-regulated *RIPK3* expression. (**C**) Cluster III was hypomethylated in 12 LUAD samples; hypomethylation at this site correlated with lower *RIPK3* mRNA levels, indicating a positive association between methylation and gene expression at this site. (**D**) Schematic representation of the CpG site positions within the *RIPK3* locus relative to annotated regulatory elements. The promoter region is in red, and the enhancers are in green. CpG sites are color coded on the basis of correlation with *RIPK3* expression: red for positive, blue for negative, and grey for no significant correlation. Correlations were computed using Pearson’s correlation.

Four hundred fifty-four LUAD cases and 21 healthy tissue samples had DNA methylation and mRNA expression profiles available. We analyzed 13 individual CpG sites in the *RIPK3* region, of which eight sites (positions 4 to 10) were located within the annotated promoter (EH38E1703809) and enhancer regions (EH38E1703807 and EH38E1703808) of *RIPK3*, while two CpG sites (positions 12 and 13) were located upstream of these regulatory regions, and three sites (1 to 3) were located outside of these elements within the gene body (Fig. 2D and fig. S2A).

CpG methylation status and *RIPK3* expression were significantly associated with 9 of the 13 CpG sites (positions 4 to 11 and 13) (figs. S2, C to O). Seven of these 10 CpG sites (positions 5 to 11) were located within the promoter and enhancer regions and defined as a CpG cluster, showing consistent methylation profiles across patients. In contrast, CpG sites 4 and 13 showed no significant correlation with CpG sites 5 to 11 or any other CpG site within the *RIPK3* region. Due to their distinct methylation patterns, they were not grouped with the central CpG cluster. Instead, each was classified as an individual CpG site forming a single-site cluster, reflecting its independent behavior (fig. S2B).

On the basis of these patterns, we identified three distinct methylation mechanisms: cluster I (CpG site 4), cluster II (CpG sites 5 to 11, forming the primary CpG cluster within the promoter-enhancer region), and cluster III (CpG site 13). Samples were classified as hypomethylated or hypermethylated at a given CpG site if their methylation levels deviated by ±0.2 from the mean of normal samples (more details in Methods). Cluster I was hypermethylated in 49 of the 454 LUAD samples, showing significantly lower levels of *RIPK3* expression [hypermethylated samples, *RIPK3* expression (RNA-Seq by Expectation-Maximization; RSEM) mean = 162.73; and unchanged methylation samples, *RIPK3* expression (RSEM) mean = 320.10; *P* < 0.01, Mann-Whitney rank sum test] (Fig. 2A and fig. S2F). Cluster II was hypermethylated in 13 of the 454 samples, with hypermethylated samples showing reduced *RIPK3* expression [hypermethylated samples, *RIPK3* expression (RSEM) mean = 98.39; and unchanged methylation samples, *RIPK3* expression (RSEM) mean = 309.20; *P* < 0.01, Mann-Whitney rank sum test] (Fig. 2B and fig. S2, G to M). Cluster III was hypomethylated in 12 of the 454 samples (Fig. 2C and fig. S2O). Cases with low methylation levels at this site exhibited significantly lower *RIPK3* mRNA expression [hypomethylated samples, *RIPK3* expression (RSEM) mean = 133.15; and unchanged methylation samples, *RIPK3* expression (RSEM) mean = 307.78; *P* < 0.01, Mann-Whitney rank sum test], indicating a positive correlation between methylation and gene expression.

Notably, the epigenetic regulation of *RIPK3* expression was not limited to the hypermethylation of the promoter. Still, it was also associated with the hypomethylation of at least one upstream CpG site, suggesting the existence of alternative regulatory mechanisms. Changes in methylation of these three CpG clusters were almost entirely mutually exclusive (fig. S2P), and most patients showed only one of the three patterns described above. In total, aberrant methylation correlating with reduced expression levels of *RIPK3* was observed in 60 of the 454 patients (13.2%).

As functional silencing of necrosome signaling can occur through the repression of *RIPK3* or the elevated expression of the negative necrosome regulator *CASP8*, we investigated the methylation and expression patterns of *CASP8* in samples from patients with lung cancer. Compared to healthy controls, we identified a substantial up-regulation of *CASP8* throughout all clinical stages in patients with LUAD [mean expression in tumor samples, log_2_(RSEM) = 9.68; mean expression in normal samples, log_2_(RSEM) = 9.32; *P* < 0.01] (fig. S3, A and B). Moreover, changes in DNA methylation correlating with overexpression of *CASP8* were observed in 421 of the 454 patients (92.73%) (fig. S3, C to J). These data suggest that the repression of inflammatory necrosome signaling results from a concomitant up-regulation of *CASP8* and a down-regulation of *RIPK3*, thereby reducing the inflammatory profile of the LUAD lesions and, consequently, the recruitment of immune cells.

The downstream molecular effector of RIPK3 for necroptotic cell death is a MLKL pseudokinase. To investigate alterations in *MLKL*, we examined its expression levels in patients with LUAD. We observed a slight down-regulation in mRNA levels (mean expression in tumor samples = 8.36 and mean expression in normal samples = 8.63, *P* = 0.02) in paired LUAD samples but no differences in *MLKL* expression across the clinical stages of LUAD (fig. S4, A and B). Moreover, protein levels proved low to weak through LUAD samples and controls, supporting that MLKL repression is not a relevant mechanism for repressing inflammation in LUAD (fig. S4, C to E). These data showed that virtually every patient with LUAD in our cohort had changes in DNA methylation, which correlated with the epigenetic silencing of inflammatory necrosome signaling, underscoring that a functional relevance of this pathway as a tumor suppressive mechanism may exist in LUAD.

### *Ripk3* deletion alters the tumor immune microenvironment

We have demonstrated that necrosome signaling components are suppressed in histologically and clinically advanced lung cancer lesions. Although genetic mutations did not explain the decreased expression of *RIPK3* in LUAD, abnormal methylation of *RIPK3* was associated with lower expression levels, a characteristic linked to poorer overall survival. To investigate the functional effects of necrosome signaling, we explored its role in tumor progression and its influence on the immune microenvironment using two well-controlled mouse models of LUAD.

**Fig. 3.**
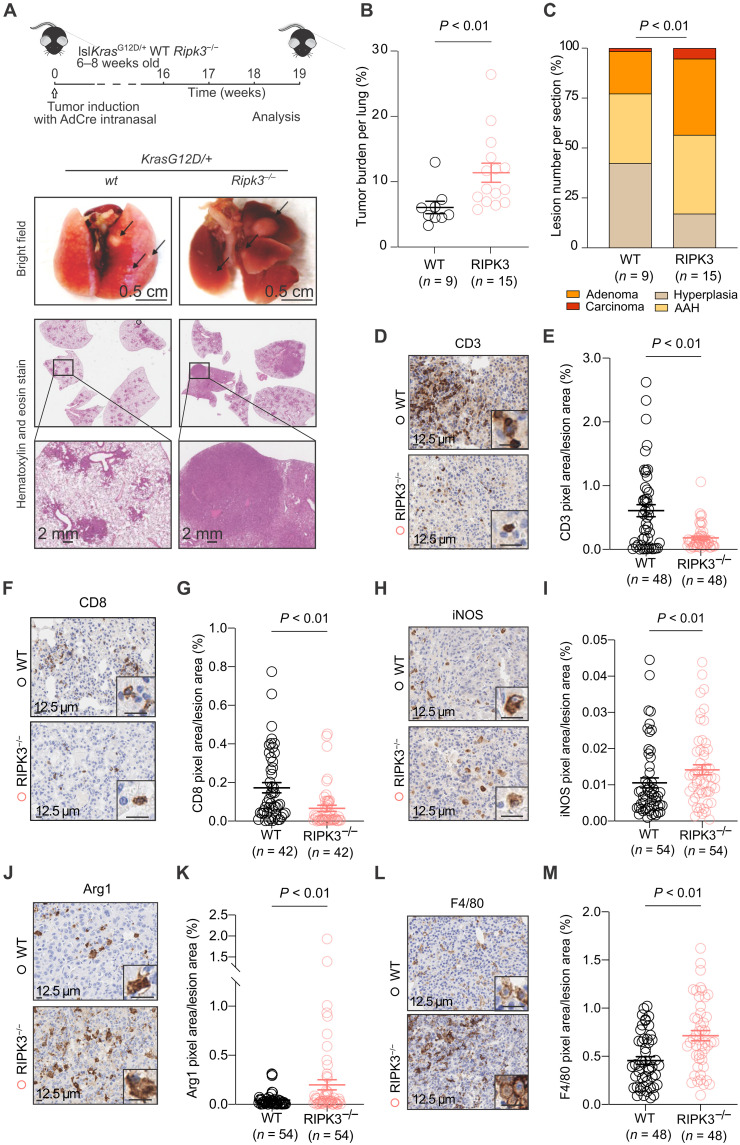
*Ripk3* deletion exacerbates tumor burden and alters the tumor immune microenvironment in oncogene-induced models of lung cancer. (**A**) Experimental design for AdCRE administration in *Kras*^*G12D*^ animals and representative images of macroscopic (top) and hematoxylin and eosin (H&E) staining of sections (bottom) of a lung from the experimental mice. Arrows mark lesions. Insets show higher magnification of the corresponding images. Scale bars are reported in the figure. (**B**) Quantification of the H&E staining is expressed as the area of lesions over the total lung area. Three sections per animal, separated by 100 μm, were assessed. *n* = number of animals. (**C**) Percentages of histologically graded tumor lesions from mice of the indicated genotypes at 19 weeks postinfection. Representative images and quantification of (**D** and **E**) CD3^+^ signal, (**F** and **G**) CD8^+^ signal, (**H** and **I**) inducible nitric oxide synthase (iNOS)–positive signal, (**J** and **K**) arginase 1^+^ signal, and (**L** and **M**) F4/80^+^ signal in lesions from *Kras^G12D/+^Ripk3^+/+^* and *Kras^G12D/+^Ripk3^−/−^* animals. Three sections per mouse were analyzed. *n* = number of animals. Data in (B), (E), (G), (I), (K), and (M) are presented as means ± SEM, analyzed by the Mann-Whitney test. Data in (C) was analyzed using Fisher’s exact test. AAH, adenomatous hyperplasia.

To clarify the function of RIPK3 signaling in a Kras-driven cancer development with reduced levels of inflammation caused by carcinogens, we used mice that carry a gain-of-function mutation in *Kras* (*Kras^G12D^*) at the endogenous location (*lox-STOP-lox-Kras^G12D^* or *lsl-Kras^G12D/+^*) and crossed them with *Ripk3^−/−^* mice (Fig. 3A). In these experimental models, the expression of the mutant *Kras* is triggered through the intranasal introduction of adenoviral CRE (AdCRE) (*30*), resulting in the random activation of the *Kras^G12D^* oncogene in specific pulmonary epithelial cells, primarily leading to adenoma formation (*31*). To analyze the impact of RIPK3 on tumor development, we quantified the number and the histologic grading of tumor lesions ranging from hyperplasia to adenocarcinoma. We detected a more advanced histological grading in the *Ripk3^−/−^* background (*P* < 0.01, Fisher’s exact test) (Fig. 3, A to C). Neither changes in proliferation nor apoptosis accounted for the differences in tumor burden, as we detected no differences in Ki-67 levels and virtually no positive cells for cleaved caspase-3 (fig. S5, A to C). Furthermore, loss of *Ripk3* did not affect the histological features of the malignant lesions, such as expression of club cell secretory protein 10, pro-surfactant C (SP-C), or mucin (fig. S5, D to I).

Our histologic survey indicated that loss of *Ripk3* was associated with an altered immune cell composition in neoplastic lesions. Specifically, we found a significant decrease in CD3^+^ [wild type (WT) = 0.45 and *Ripk3*^−/−^ = 0.11; *P* < 0.01, Mann-Whitney rank sum test] and CD8^+^ (WT = 0.09 and *Ripk3*^−/−^ = 0.01; *P* < 0.01, Mann-Whitney rank sum test) T and B lymphocytes in *Ripk3^−/−^* mouse lesions (Fig. 3, D to G). We also identified elevated macrophages skewed toward protumoral subpopulations (Fig. 3, H to K) (arginase 1–positive, M2-like differentiated). To exclude an intrinsic deficiency in the immune cells of *Ripk3*-deficient animals, we analyzed the healthy lungs of *Ripk3^−/−^* and WT mice. We detected no overt differences between genotypes in these immune cell subsets (fig. S6). This showed that loss of RIPK3 reduced the number and complexity of tumor-associated immune cell populations such as cytotoxic T cells (Fig. 3F).

### *Ripk3* tumor-specific deletion exacerbates tumor burden

**Fig. 4.**
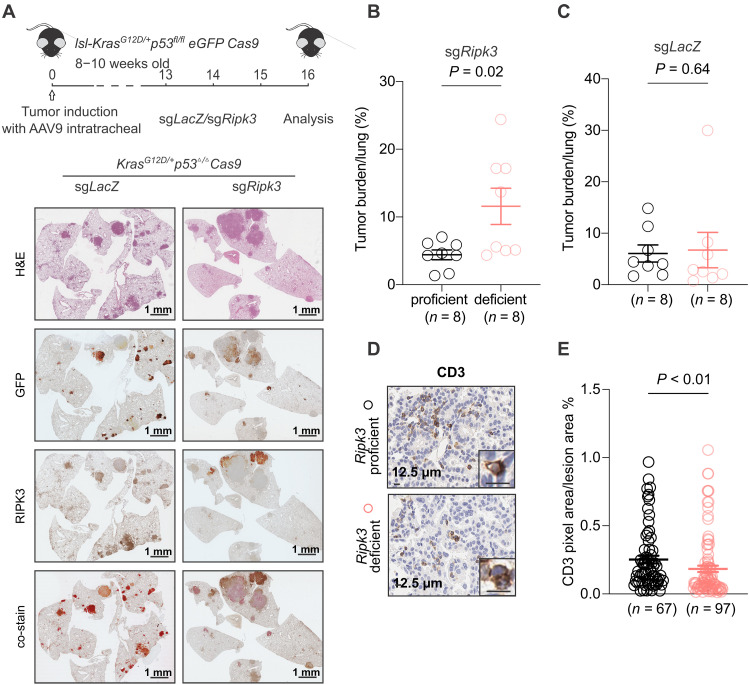
*Ripk3* tumor-specific deletion exacerbates tumor burden. (**A**) Experimental design for AAV9 administration in *lsl-Kras^G12D^;p53^fl/fl^;Cas9* animals and representative images of H&E, GFP, and RIPK3 in staining (as indicated) of lung from the experimental mice in the costain slide; red indicates GFP^+^ cells, and brown indicates RIPK3^+^ cells of tumor cells. Scale bars are reported in the figure. (**B** and **C**) Quantification of the H&E staining expressed as area of lesions over lung area for GFP-positive versus GFP-negative lesions in sg*Ripk3* (B) and sg*LacZ* (C) animals. Three sections per animal, separated by 100 μm, were assessed. *n* as reported in the figure. (**D**) Representative images of the CD3^+^ signal in lesions from experimental animals. (**E**) Quantification of CD3^+^ signal. Data in (B), (C), and (E) are presented as means ± SEM. Data in (B) and (C) were analyzed using the Wilcoxon signed-rank test (paired). Data in (E) were analyzed using the Mann-Whitney test.

To address the complicating factors associated with total body knockout mice, we used CRISPR-Cas9 gene editing in vivo. We used mice that contain lsl-Cas9 in the Rosa26 locus (*32*) and crossed them with mice of the *lsl-Kras^G12D^Tp53^fl/fl^* background. We introduced CRE recombinase and the single guide RNA (sgRNA) for *Ripk3* via intratracheal instillation of adeno-associated virus (serotype 9, AAV9). This process of CRE recombination activated *Kras*^*G12D*^ and Cas9 expression, confirmed by green fluorescent protein (GFP) coexpression (*Cas9-P2A-EGFP*) and *Tp53* deletion (fig. S7). Concurrently, sgRNA enabled the selective deletion of *Ripk3*, specifically in the transformed cells. The loxP-flanked *p53* mice were used to model a more aggressive phenotype, mimicking that of human patients with LUAD. We designed a tailored and effective sgRNA for *Ripk3*, validated it in vitro, encapsulated it within the AAV9 capsid, and assessed its efficacy in vivo. For the negative control, sgRNA targeting LacZ was used. We administered AAV9-sg*Ripk3* or AAV9-sg*LacZ* through intratracheal injection to 8- to 10-week-old mice and evaluated tumor burdens at week 16 (Fig. 4A). We observed a heterogeneous phenotype across the animals. CRE recombination demonstrated incomplete penetrance in both cohorts, as indicated by tumor lesions with and without Cas9 activation within the same specimen (Fig. 4A). We leveraged this mosaicism to examine tumor burden and immune cell infiltration in *Ripk3*-proficient and *Ripk3*-deficient lesions side by side within the same experimental animal (Fig. 4A).

Consistent with findings from total body knockout models, we observed an elevation in tumor burden for RIPK3-negative compared to that for GFP-positive lesions (GFP negative = 4.49; and GFP positive = 9.64; *P* = 0.02, Mann-Whitney rank sum test), representing those with a complete genetic deletion of *Ripk3* (Fig. 4, B and C). To eliminate the possibility of bias from CRE influencing this outcome, we compared GFP-positive and GFP-negative lesions in the sg*LacZ* control group and detected no differences (fig. S7). Additionally, we assessed immune cell infiltration within the sg*Ripk3* cohort by comparing GFP-positive and GFP-negative lesions in the same animal, which depict *Ripk3*-deficient and *Ripk3*-proficient lesions, respectively. In the sg*Ripk3* group, there was a noticeable decrease in T lymphocyte infiltration in *Ripk3*-deficient lesions (*Ripk3* proficient = 0.17 and *Ripk3* deficient = 0.08; *P* < 0.01, Mann-Whitney rank sum test), reinforcing the finding that RIPK3 signaling plays a role in promoting T cell infiltration (Fig. 4, D and E).

### *RIPK3* expression is associated with an elevated immune cell infiltration in human LUAD

After confirming that RIPK3 functions as a genuine tumor suppressor in lung cancer, which is linked to a significant decrease in immune cell infiltration into the tumor microenvironment, we aimed to connect these findings to two separate transcriptomic analyses of human lung cancer. This is based on the hypothesis that necroptosis could influence tumor immunogenicity by releasing cytokines and damage-associated molecular patterns. To this end, we analyzed transcriptomic data originating from the TCGA and TRACERx datasets (*25*–*27*).

**Fig. 5.**
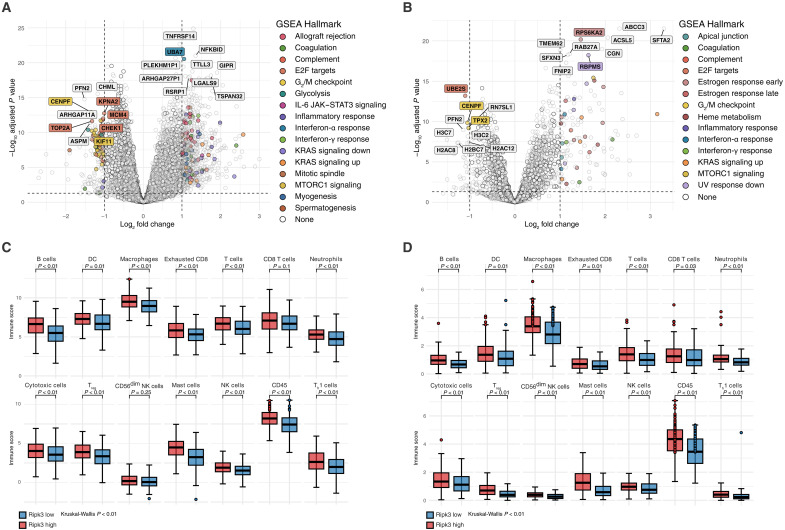
*RIPK3* expression is associated with an elevated immune cell infiltration in human LUAD. (**A** and **B**) Differential gene expression in TCGA-LUAD (A) and TRACERx (B) samples with RIPK3^hi^ expression versus RIPK3^lo^ expression, as defined by upper and lower quartiles. Significantly differentially expressed genes are color coded by their associated Hallmark term (only one Hallmark term per gene is shown, selected on the basis of the highest normalized enrichment score (NES) score from GSEA). *RIPK3* itself is not shown. (**C** and **D**) Association of *RIPK3* expression (upper quartile versus lower quartile) and the abundance of immune cell populations (immune score) in TCGA-LUAD (C) and TRACERx (D) cases using the method of Danaher *et al.* (*33*). Mann-Whitney test, FDR correction. JAK, Janus kinase; STAT3, signal transducer and activator of transcription 3; UV, ultraviolet; NK, natural killer.

In TCGA, differential expression analysis identified 652 up-regulated (with log fold change (*LFC*) ≥ 1 and false discovery rate (FDR < 0.05) and 171 down-regulated genes (with *LFC* ≤ −1 and FDR < 0.05) in RIPK3^hi^ tumors compared to those in RIPK3^lo^ tumors (RIPK3^lo^ = 129 and RIPK3^hi^ = 129, as defined by quartiles of *RIPK3* gene expression) (Fig. 5A). Gene set enrichment analysis (GSEA) using Hallmark and Gene Ontology Biological Process gene sets revealed a consistent enrichment of immune-related pathways in RIPK3^hi^ tumors (Fig. 5A and fig. S8, A, B, and E). Specifically, Hallmark gene sets such as allograft rejection, complement, IL-6–JAK–STAT3 Signaling, interferon-γ response, interferon-α response, and inflammatory response were significantly enriched, along with KRAS signaling (both up- and down-regulated components), suggesting the engagement of innate and adaptive immune programs (Fig. 5A and fig. S8B). Complementary Gene Ontology Biological Process (GO:BP) analysis further supported this immune-activated phenotype, highlighting enrichment of leukocyte cell-cell adhesion, regulation of immune effector processes, leukocyte-mediated immunity, and adaptive immune response (fig. S8, C and E). In contrast, RIPK3^lo^ tumors exhibited positive enrichment of proliferative and biosynthetic pathways, including E2F targets, G2/M checkpoint, MYC targets, mTORC1 signaling, oxidative phosphorylation, and glycolysis. Corresponding GO:BP terms such as mitotic chromosome segregation, DNA replication, ribosome biogenesis, and cell cycle phase transitions were also enriched, indicating a proliferative, metabolically active transcriptional state in RIPK3^lo^ tumors (fig. S8).

We validated these findings by using an independent dataset of samples from patients with LUAD obtained from the TRACERx lung cohort (548 samples from 217 patients). We identified 122 up-regulated and 43 down-regulated genes (*LFC* ≥ 1 or ≤ −1 and FDR < 0.05) in RIPK3^hi^ versus RIPK3^lo^ tumors (*n* = 134 per group) (Fig. 5B). Consistent with findings from TCGA, RIPK3^hi^ tumors showed significant enrichment of immune and inflammatory Hallmark gene sets, including complement, inflammatory response, TNFA signaling via nuclear factor κB, and interferon-γ response, alongside GO:BP terms such as cell adhesion, supporting a stromal and immune-activated phenotype (Fig. 5B and fig. S8, C, D, and F). In contrast, RIPK3^lo^ tumors in both datasets were enriched for proliferative and metabolic programs, including E2F targets, MYC targets, oxidative phosphorylation, and mTORC1 signaling, indicating a biosynthetically active state (Fig. 5B and fig. S8, C, D, and F). Notably, several immune-related genes were consistently up-regulated in RIPK3^hi^ tumors across both TCGA and TRACERx, including *CIITA*, *CXCL17*, *PIGR*, *IFI27*, *UBA7*, and *DPP4*, indicating enhanced antigen presentation, chemokine signaling, and interferon responses. Together, these results suggest an association between high *RIPK3* expression and immune activation in NSCLC.

Given these results, we next evaluated immune cell infiltration in tumor lesions of patients with LUAD on the basis of immune signatures described by Danaher and colleagues (*33*), which estimate immune cell subpopulations in tumor lesions using mRNA counts of immune cell–specific marker genes. In line with the GSEA, we observed generally higher expression of immune cell markers in the RIPK3^hi^ cohort (Fig. 5C). Specifically, we detected a significant increase in gene expression signatures representing various subsets of T and B lymphocytes, including the total number of tumor-infiltrating lymphocytes. Myeloid immune cells, such as macrophages, mast cells, neutrophils, and dendritic cells, were also more prevalent in tumor lesions with high *RIPK3* expression. We validated these findings in TRACERx, confirming that elevated *RIPK3* expression levels correlated with a rich immune environment in patients with LUAD (Fig. 5D).

## DISCUSSION

Avoidance of cell death is a critical survival strategy during oncogenic transformation (*4*). Accordingly, many tumors exhibit genomic amplifications of anti-apoptotic genes or loss of proapoptotic proteins, allowing them to evade regulated cell death programs (*4*). Furthermore, small-molecule inhibitors such as ABT-199, which promote apoptosis, have shown substantial clinical benefits (*34*). While apoptosis is essential in lung cancer onset and persistence, the role of necroptosis in lung cancer is not clearly understood. In addition, the advent of immune checkpoint inhibitors and, more broadly, engagement of the immune system to combat cancer have changed the focus from apoptosis to immunogenic types of regulated cell death, such as necroptosis. As such, previous studies from our group and others have revealed a strong tumor-suppressing function of RIPK3-mediated cell death across multiple cancer types and oncogenic drivers (*21*, *22*, *35*). However, RIPK3 is also being increasingly recognized for mediating non–cell-death signaling functions, including proinflammatory signaling independently of necroptosis (*18*, *19*, *36*). This is highlighted by RIPK3’s activation of the NLRP3 inflammasome pathway, which leads to interleukin-1β (IL-1β) production (*37*, *38*). Here, we systematically interrogated the role of RIPK3 in LUAD. Our findings indicate a positive correlation between *RIPK3* expression and inflammatory markers in human LUAD lesions. We demonstrate that in vivo deletion of *Ripk3* exacerbates cancers with *Kras* mutations, either alone or in combination with *Tp53* deletion, which was associated to alterations in the immune landscape. This role differs from past studies, which established RIPK3 as a tumor promoter in *Kras*-driven pancreatic cancer (*22*), and argues for a nuanced context-dependent role of necroptosis in cancerogenesis.

Furthermore, lung epithelial cells often interact with pathogens, enabling them to activate antimicrobial pathways, which may include necroptosis but could also involve necroptosis-independent signaling pathways. Consistent with this interpretation, our data do not provide direct evidence that the main course of action of RIPK3 in LUAD is via necroptotic cell death. The observed increase in Caspase 8 (CASP8) and the lack of correlation with MLKL expression suggest that cell-death–independent functions of RIPK3 may predominate in this context (*37*). Together, these observations extend RIPK3’s roles in LUAD beyond its canonical function as a mediator of necroptosis.

Contextual carcinogenesis, influenced by cell type, results in distinct phenotypes even with the same oncogenic driver mutation. For instance, *KRAS* or *PIK3CA* mutations produce distinct tumors with variations in morphology, invasiveness, and aggressiveness based on their cell of origin. Our findings align with studies showing that the antitumor properties of RIPK3, with high RIPK3 levels linked to better outcomes, such as in the study by Li *et al.* (*39*), which investigated the RIPK family in LUAD and found RIPK3 to negatively correlate with prognosis, underlining its clinical relevance. No recurrent genetic mutations or chromosomal abnormalities affect *RIPK3* in lung cancer, highlighting the identified control by epigenetic regulation. We found that methylation changes near the *RIPK3* promoter reduced the transcription of *RIPK3* in LUAD. Three distinct methylation patterns reduce *RIPK3* transcription, indicating that targeted analysis of individual CpG methylation is critical.

Nevertheless, *RIPK3* silencing in LUAD is likely multifactorial, and aberrant methylation is an important, but not exclusive, mechanism of *RIPK3* repression. For example, histone modifications can silence gene expression independently of DNA methylation (*40*), and transcription factors such as SP1 or FOXA2 have been shown to regulate *RIPK3* expression in a context-dependent manner (*41*). These layers are not captured in the current dataset but remain compelling areas for future investigation. Last, while we identified both hypermethylation and hypomethylation associated with *RIPK3* expression, the upstream drivers of these methylation patterns remain unknown. Future integrative analyses incorporating chromatin accessibility, histone mark profiling, and transcription factor occupancy will be essential to fully elucidate the regulatory landscape governing *RIPK3* expression in LUAD.

A recent report using single-cell RNA sequencing (RNA-seq) in a breast cancer mouse model corroborates our proposed mechanism. Late-stage tumors were found to suppress immune-related genes, especially those involved in interferon signaling, as well as genes critical for the induction of necroptosis and pyroptosis, notably *Ripk3*, leading to immune evasion. By inhibiting DNA methylation using low-dose decitabine, this suppression could be impeded, which, in turn, restored antitumor immunity and reduced tumor growth in breast and melanoma models (*42*). Similarly, the repression of the propensity to undergo necroptosis by restricting *RIPK3* expression was shown in AML (*21*, *43*), mesothelioma (*44*), liver disease (*45*), and colorectal cancer (*46*). These data are of particular interest in our model, as similar effects were observed in epithelial lung cancer cells, suggesting that hypomethylating agents may be a sensible therapeutic strategy in a wider array of solid cancers (*42*). Thus, we demonstrate that epigenetic silencing enables cancer cells to evade the tumor-suppressive and immune-modulating effects of RIPK3 in LUAD. Our results highlight the related RIPK3-dependent signaling as a key link between abnormal DNA methylation patterns, inflammatory profiles, and immune cell infiltration, thereby bridging the immune microenvironment and epigenetics in lung cancer. Reactivating RIPK3 activity may enhance immune responses and tumor cell recognition in patients with pulmonary adenocarcinoma.

Aberrant inflammation is being recognized as a key driver in the pathogenesis of LUAD (*47*, *48*). For instance, epidemiological data implicate air pollutants in elevating lung cancer risk, in part, via dysregulated IL-1 signaling (*49*). Furthermore, post hoc analyses of the CANTOS trial (*50*), initially designed to assess IL-1β blockade in cardiovascular disease, revealed a significant reduction in lung cancer incidence among treated individuals. These reports indicate that elevated inflammation in the tumor environment promotes the development of lung cancer. However, inflammation also elicits necrosome formation (*6*–*9*, *38*, *39*), prompting the need for tumor cells to suppress necroptotic cell death and T cell–mediated immunity. Our data now delineate a mechanism by which tumor cells protect themselves against RIPK3-mediated tumor-suppressive signaling, including necrosome activation, thereby supporting continued tumor progression. We identify epigenetic silencing of *RIPK3* as a critical event and demonstrate that impaired RIPK3 function results in reduced T cell–mediated tumor control. These findings suggest that reengaging RIPK3-dependent pathways, e.g., via epigenetic modulation, may represent a transformative approach to targeting inflammation-associated tumor progression.

### Limitations of this study

While the present study offers important insights into the role of RIPK3 in LUAD, several limitations warrant consideration, each of which may represent an opportunity to extend this line of research. First, although we used multiple genetically induced mouse models to dissect the role of RIPK3 in lung cancer, these systems are not able to entirely capture the complexity and heterogeneity of human LUAD. Moreover, our study investigates the repression of RIPK3 and its ensuing effects on immune cell infiltration as well as tumor progression. However, necroptosis involves many more pathway members, such as RIPK1, MLKL, or CASP8. Scrutiny of their individual contribution will be crucial to obtain a comprehensive understanding of necroptosis in lung cancer. Thus, while our study identifies a clear association between RIPK3 deficiency, enhanced tumor growth, and reduced immune infiltration, the mechanistic link between these observations remains unresolved. Specifically, we cannot now distinguish whether RIPK3 loss confers a tumor-intrinsic growth advantage or primarily facilitates immune evasion. Addressing this will require targeted immune cell depletion or lineage-specific deletion models, which lie beyond the scope of this study but are the focus of ongoing work. Our dataset also does not allow us to distinguish whether cell death-independent pathways contribute to the observed effects or whether RIPK3-mediated necroptotic cell death is involved. Last, while we identified methylation changes in the *RIPK3* promoter region associated with reduced expression, the upstream regulators determining these methylation patterns remain uncharacterized. Neither have we examined other epigenetic mechanisms, such as histone modifications or chromatin accessibility, that may contribute to *RIPK3* repression.

## METHODS

### TCGA data preprocessing

Preprocessed gene expression levels of LUAD cases were downloaded from TCGA Genomics Data Commons Portal. Somatic variant calls, CNVs, and DNA methylation beta values were accessed through the Xena portal (*51*). Gene expression counts were based on RSEM values; somatic variants were used as detected by “Mutect.” CpG sites with “NA” beta values for all samples, CpG sites containing “NaN” values, CpG sites with gender-specific methylation (Spearman’s rank correlation coefficient ≥ 0.7), and cross-reactive CpG sites were excluded from further analysis. Samples were defined as “tumor samples” on the basis of their annotation as “primary tumor.” Patients with unknown survival (“vital_status” or “days_to_death”) have been excluded from the survival analysis.

### TRACERx data preprocessing

Multiregional gene expression raw data from TRACERx samples were downloaded through the secure file transfer protocol (SFTP) command line and decrypted using crypt4gh. Samples were processed using nf-core/rnaseq (Nextflow 24.10.4, nf-core/rnaseq v3.18.0) pipeline (*52*), including quality assessment using fastqc, mapping of the reads to human reference genome version GRCh38 (hg38) using STAR (v2.7.11b), and gene expression was quantified using Salmon (v1.10.3). Overall, 85 normal samples from 85 patients and 808 tumor samples from 324 patients were available for analysis. After excluding lung squamous cell carcinoma (LUSC) samples, 548 samples from 217 patients were used for further studies.

### CPTAC data preprocessing

CPTAC-LUAD proteomics, transcriptomics, and clinical data were downloaded via LinkedOmicsKB (*53*). Gene expression data for both tumor and normal samples were represented as upper-quartile normalized log_2_-transformed RSEM values (LUAD_RNAseq_gene_RSEM_coding_UQ_1500_log_2_). Proteomic data were obtained as log_2_-transformed, reference-intensity–normalized protein abundances (LUAD_proteomics_gene_abundance_log_2__reference_intensity_normalized). For the analysis, only patients with paired tumor and normal samples (*n* = 101) were included. Patients with unknown survival (“OS_event” or “OS_days”) have been excluded from the survival analysis.

### Lehtiö proteomics data preprocessing

Proteomic data were obtained from the study by Lehtiö *et al.* (*28*), comprising 141 tumor samples, using the DDA proteomics dataset provided in their supplementary materials (table 2, “Table2-DDAProteomics”). Protein abundance values from this table were used directly for downstream analyses. Clinical information, including overall survival, was extracted from the same source (table 1, “Table1-EarlyStageCohortMetadat”). For survival analyses, patients with unknown information for the survival outcome variable (“OSbin”) or with unknown overall survival time defined as “OS_years” were excluded from the analysis.

### CNV and SNV analysis

Single nucleotide variants (SNVs) were accessed from the TCGA Unified Ensemble “MC3” mutation calls. CNVs were defined on the basis of Genomic Identification of Significant Targets in Cancer (GISTIC) thresholded data to identify samples with significant CNVs. We focused on high-confidence alterations, corresponding to GISTIC scores of ±2, representing deep deletions and high-level amplifications.

### Methylation analysis

A curated panel of 13 *RIPK3*-associated CpG sites (cg27599271, cg13583230, cg20038493, cg20822579, cg13050240, cg13066043, cg11781986, cg23621631, cg13796295, cg23038074, cg27175788, cg10318258, and cg00951869) was selected for analysis. Each CpG site’s beta value was tested separately for correlation with expression to identify CpGs most strongly associated with *RIPK3* transcriptional activity. A correlogram was constructed to explore methylation clustering and inter-CpG relationships using Pearson correlation coefficients across beta values of the CpGs. The CpGs were ordered on the basis of genomic coordinates to assess local methylation patterns and potential comethylation blocks. After identifying three separate methylation clusters, the mean beta value across CpGs in the cluster was calculated for each tumor sample. Tumor samples were classified as hypermethylated, if their mean beta value exceeded the median of matched normal samples by ≥0.2, and hypomethylated, if their mean beta value was below the median by ≥0.2. Otherwise, it was classified as unchanged methylation. Samples were then matched with *RIPK3* expression values, and correlation analysis was performed using Pearson’s correlation coefficient.

### Differential gene expression analysis and GSEA

Five hundred fifteen patients with TCGA-LUAD were classified into RIPK3^lo^ (129 patients) and RIPK3^hi^ (129 patients) expressing tumors using the first and fourth quartile of normalized RNA-seq RSEM values, respectively. Five hundred thirty-seven TRACERx samples were classified into RIPK3^lo^ (134 samples) and RIPK3^hi^ (134 samples) again using the first and fourth quartiles of raw counts, respectively. The analysis was run in R (v4.4.1). Differential gene expression analysis was performed using the limma-voom pipeline from the limma (v.3.60.6) and edgeR (v.4.2.2) packages. Lowly expressed genes were filtered using filterByExpr() to retain only genes with sufficient counts across samples. The resulting filtered dataset was normalized using the trimmed mean of *M*-values normalization via calcNormFactors(). Gene-wise variance modeling was conducted using the voom transformation. A design matrix specifying the experimental groups (e.g., *RIPK3* high versus low expression) was applied to fit the linear model using lmFit(). For the TCGA dataset, a standard linear model was fitted, and empirical Bayes moderation of standard errors was applied using eBayes() to obtain robust statistics. For the TRACERx dataset, which includes multiple tumor regions per patient, interpatient correlation was accounted for using duplicateCorrelation(). The model was then fitted with a block argument (patient ID) and estimated consensus correlation, allowing repeated measurements to be adjusted. GSEA was conducted using the fgsea (v1.30.0) package to identify biological pathways associated with differential expression patterns between *RIPK3* high and low samples. Gene sets for analysis were obtained from the MSigDB collection using the msigdbr (v25.1.1) package.

### Immune cell deconvolution

For immune evaluation, the method of Danaher *et al.* was used (*33*). The evaluated immune cell populations were CD8^+^ T cells (“CD8”), exhausted CD8^+^ T cells (“CD8 exhausted”), CD4^+^ T cells (“CD4”), regulatory T cells (“T_reg_”), helper T cells (“T_H_1”), dendritic cells (“DC”), B cells (“B cells”), neutrophils, macrophages, CD45^+^ cells (“CD45”), and measures for total T cells (“T cells”), total tumor-infiltrating lymphocytes (“total TIL”), and cytotoxic cells (“cyto”).

### Human patient samples and tissue analysis

Eleven primary LUAD and matched health tissues were stained against RIPK3 (1:1000, ProSci, 2283; 8337-1603). Tissue microarray (TMA) was constructed using 25 samples from patients with LUAD from the Tissue bank from University Hospital of the Technical University of Munich (MRI, TUM) (ethical approval no. 553/15 S). Hematoxylin and eosin (H&E)–stained slides were used to avoid areas of necrosis and to define two peripheral and one central tumor areas, which were marked on the donor blocks. Formalin-fixed paraffin-embedded tumor samples were assembled into a TMA using a Tissue Microarrayer, extracting 1-mm-diameter cores from the three separate predefined areas. Sections (2 μm) were cut with a microtome and stained with the RIPK3 and MLKL antibody [1:1000 (ProSci, 2283; 8337-1603) and 1:500 (Abcam, ab184718), respectively] and counterstained with hematoxylin using an automated immunostainer with the Bond Polymer Refine Detection kit. Expression (for matched samples and TMA) was semiquantitatively measured, and the immune reactive score was calculated, consisting of staining intensity (0 to 3) and percentage of positive cells. Scores were assessed by senior pathologists blinded to the diagnosis, taking alveolar macrophages as a reference for strong staining, as they showed diffuse and distinct positivity throughout all cases.

### Mice

Previously established *lox-STOP-lox-Kras^G12D^* (*lsl-Kras^G12D^*) (*lsl-Kras*^G12D/+^; stock no. 008179; RRID: IMSR_JAX:008179), Rosa26-LSL-*Cas9* (stock no*. 024857; RRID: IMSR_JAX:024857*), *p53^fl/fl^* (stock no. 008462; RRID: IMSR_JAX:008462), and *Ripk3^−/−^* mice (stock no. 016144; RRID: IMSR_JAX:016144), all on a *C57BL/6J* background, were used to generate all the lines here. Both male and female mice, aged 6 to 8 weeks, were used for experiments. Animals were maintained under specific pathogen–free conditions and were assigned to experimental groups based solely on genotype after birth; there were no other factors determining group selection All animal experiments complied with protocols approved by the local animal ethics committee guidelines (AZ: 55.2-1-54-2532-55-12 and ROB- 55.2-2532.Vet_02-17-199) approved by the District Government of Upper Bavaria.

### CRISPR-Cas9 gene editing and validation of sgRNA

Four sgRNAs were designed against Ripk3 and LacZ using the MIT CRISPR Zhang lab CRISPR design tool (formerly available at http://crispr.mit.edu; no longer online) and cloned in pSpCas9(BB)-2A-GFP (PX458) under U6 promoter [U6-Fwd sequence (5′-3′) GAGGGCCTATTTCCCATGATTCC], a gift from F. Zhang (Addgene, plasmid no. 48138; http://n2t.net/addgene:48138; RRID: Addgene_48138), which is also coding for Cas9 and GFP. According to the manufacturers’ instructions, the plasmid was transfected in mouse embryonic fibroblasts (MEFs) using Metafectene reagent (Biontex, Munich, Germany). MEFs were grown for 2 days at 37°C in a 5% CO_2_ atmosphere in Dulbecco’s modified Eagle’s medium (Gibco), supplemented with 10% fetal calf serum (Pantech, Aidenbach, Germany) and 1% penicillin/streptomycin (Life Technologies). Cells (GFP positive) were then sorted using FACSAria II flow cytometer cell sorter (BD Biosciences, San Jose, CA, USA) and cultured as single-cell clones. Successful deletion of *Ripk3* was evaluated by Western blot. For in vivo experiments, validated sgRNA against *Ripk3* (sgRipk3_3) and control sgRNA against *LacZ* were cloned into AAV:ITR-U6-sgRNA (backbone)-pEFS-Rluc-2A-CRE-WPRE-hGHpA-ITR, a gift from F. Zhang (Addgene, plasmid no. 60226; http://n2t.net/addgene:60226; RRID: Addgene_60226) and sent for AAV9 packaging and production to Vector Biolabs (Malvern, PA, USA).

### Tumor burden analysis and grading

All lungs were sectioned into three step sections at 100-μm intervals. The 10th section of each step section was stained for H&E and used for analysis. Three H&E-stained sections per animal were analyzed. The sections were scanned with an SCN400 slide scanner (Leica Microsystems, Wetzlar, Germany). For tumor burden analysis, the total numbers of lesions on each section were counted, and the area (square micrometers) of individual lesions and total lung area (square micrometers) were determined manually using the freehand mode of the Tissue IA image analysis software (Slidepath, Leica Microsystems). For tumor grading, the tumors were graded into four primary development stages: (i) hyperplasia; (ii) atypical adenomatous hyperplasia, in which proliferation of atypical epithelial cells growing along alveolar septae takes place without disrupting the underlying lung architecture; (iii) adenomas that are neoplasms with papillary, solid, or mixed architecture that distort or obliterate the alveolar septae; and (iv) adenocarcinoma consisting of numerous mitoses and nuclear pleomorphisms including engorged nuclei with prominent nucleoli. The number of lesions was evaluated according to their development stage per mouse per square millimeter of lobe area.

### Immunohistochemistry

Immunohistochemical stainings were performed with an automated staining system, Bond Max (Leica microsystems). The following antibodies were used: Ki67 (1:200; Thermo Fisher Scientific, RM-9106 - S1), CD3-SP7 (1:50; DCS, CI597R06), CD45R-B220 (1:50; BD, 550286), F4/80 (1:50; Bio-Rad, MCA497R), MPO (1:200; LabVision Thermo Fisher Scientific Neomarkers, RB-373-A0), MLKL (1:500; Abcam, ab184718), CC10 (1:100; Santa Cruz Biotechnology, sc-9772; K0714), SP-C (1:500; Santa Cruz Biotechnology, sc-7705; K1714), cleaved caspase-3 (Asp^175^) (1:300; Cell Signaling Technology, 9661), RIPK3 (1:1000; ProSci, 2283), arginase 1 (1:600; Cell Signaling Technology, 93668), CD8 (1:100; Dianova, DIA-808), inducible nitric oxide synthase (1:250; Abcam, ab15323), and GFP (1:2000; Fitzgerald, 20R-GR011). The slides were scanned using an SCN400 slide scanner (Leica Microsystems) and analyzed using Tissue IA image analysis software (Slidepath, Leica Microsystems) and ImageScope Aperio (Leica Microsystems).

### Quantification and statistical analysis

All statistical tests were performed using R version 4.4.1 (www.r-project.org/) or GraphPad Prism. Unless otherwise mentioned, the Wilcoxon rank sum test (as implemented in wilcox.test) was used to compare groups, and *P* values were adjusted using the FDR method. In survival analyses, to avoid bias from sparse data, we truncated survival curves in each group at the last time point where more than five events remained in each group, as Cox model estimates generally require at least 5 to 10 events per covariate (*54*, *55*). This resulted in truncation times corresponding to 5 years in the TCGA-LUAD transcriptomics and methylation datasets and to 2.5 years in the CPTAC-LUAD and Lehtiö proteomics datasets. Log-rank test was used to compare Kaplan-Meier survival curves in TCGA-LUAD datasets. Firth’s penalized Cox proportional hazards model was applied to reduce small-sample bias in CPTAC-LUAD and Lehtiö datasets. Unless otherwise noted, statistical significance was considered as FDR < 0.05. Further statistical details can be found in the figure legends.
