# Supplementary material for: Aberrant methylation limits antitumoral inflammation in lung adenocarcinoma by restricting RIPK3 expression
Source: Sci Adv. 2026 Jan 21;12(4):eadz9227. doi: 10.1126/sciadv.adz9227 (PMC13249207; doi:10.1126/sciadv.adz9227)
Supplement: Supplementary file 1 — Figs. S1 to S8 Tables S1 and S2 [file sciadv.adz9227_sm.pdf]

Science Advances
AAAS

Supplementary Materials for

Aberrant methylation limits antitumoral inflammation in lung adenocarcinoma by restricting RIPK3 expression

Deepti Agrawal *et al.*

Corresponding author: Katarina Cisarova, katarina.vizar-cisarova@medunigraz.at;
Philipp J. Jost, philipp.jost@medunigraz.at

Sci. Adv. **12**, eadz9227 (2026)
DOI: 10.1126/sciadv.adz9227

This PDF file includes:

Figs. S1 to S8
Tables S1 and S2

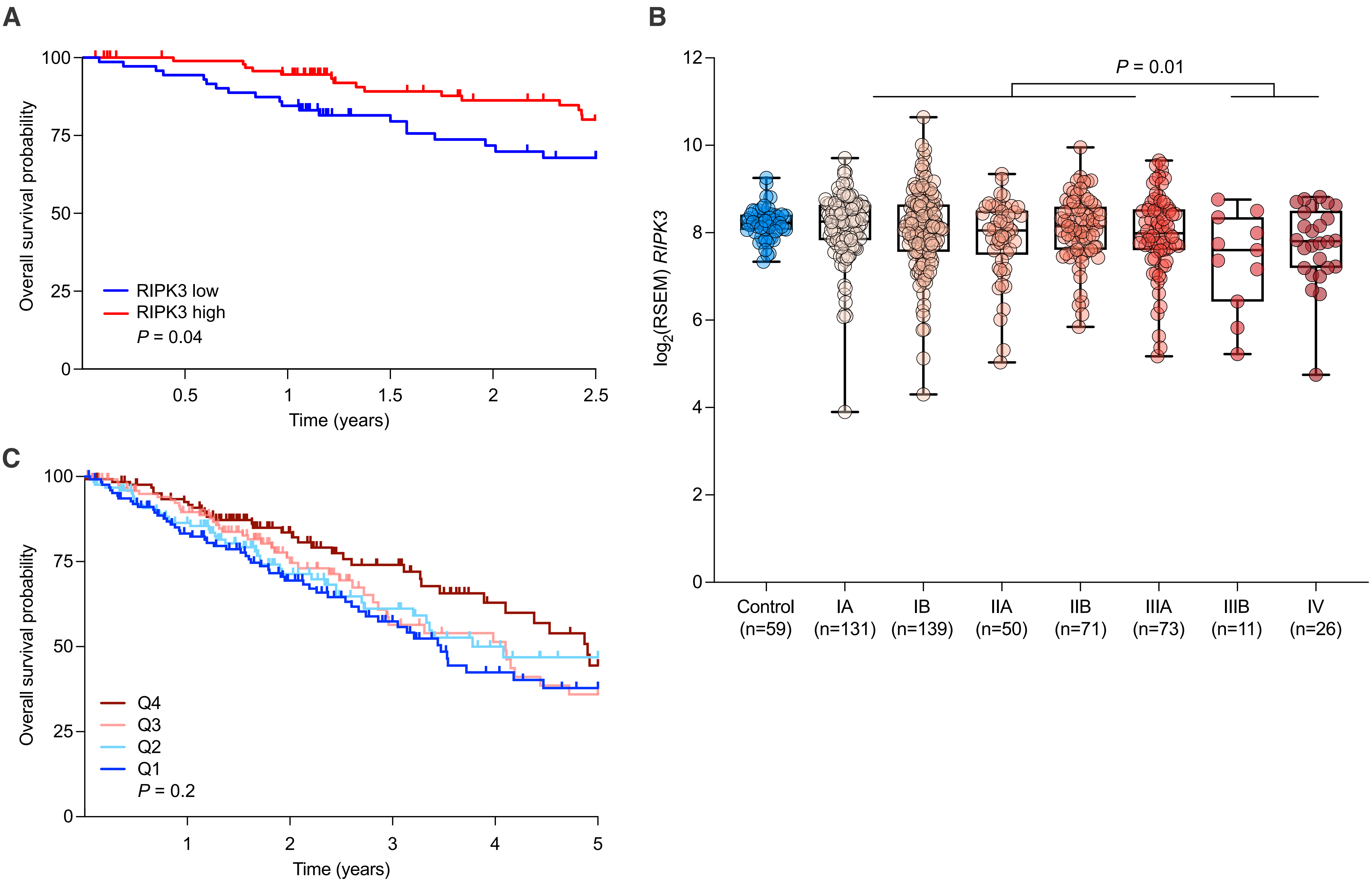

Figure S1: RIPK3 characterization in human lung cancer. (A) Kaplan-Meier plot showing the differences in overall survival in combined CPTAC and *Lehtiö* proteomicsdatasets (*28*). (B) *RIPK3* mRNA levels in TCGA-LUAD according to individual tumor stages. (C) Kaplan-Meier plot showing the differences in overall survival of TCGA-LUAD cases concerning *RIPK3* expression groups, as defined by quartiles. Firth's penalized Cox proportional hazards model was applied to data in (A). (B) was analyzed by theWilcoxon rank-sum test. (C) was analyzed by the log-rank test.

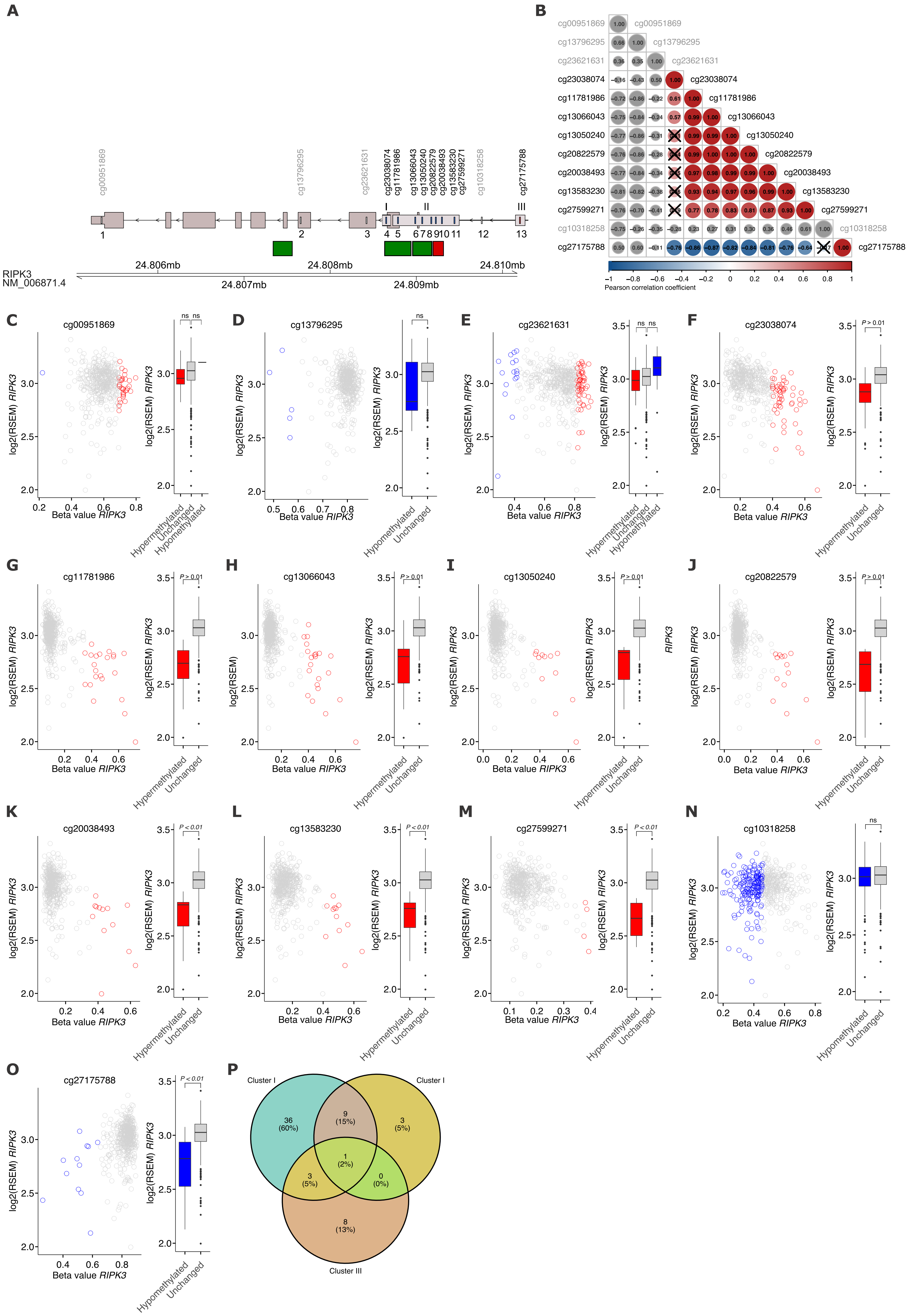

Figure S2: DNA methylation of CpG sites located in *RIPK3*. (A) Schematic representation of the gene *RIPK3*; grey boxes indicate exons; grey lines indicate introns. CpG sites along the gene are indicated as blue (if negatively correlated with *RIPK3* expression), red (if positively correlated with *RIPK3* expression) or grey (if no correlation is detected) rectangles, enhancers are indicated as green and promoters as red rectangles below the gene schema. (B) Correlogram indicating correlations between individual CpG sites. Sites in grey show no significant difference in *RIPK3* expression between samples with aberrant methylation and samples with unchanged methylation (aberrant methylation was defined as a sample deviating ±0.2 from the mean of the normal samples, for more details see *Methods*). The color and size of the dots represent the value of Pearson correlation coefficients. Crossed dots represent a non-significant *P* value of the correlation test. (C-O) Correlation between expression of RIPK3 (in log2(RSEM)) and beta values of individual CpG sites. The difference in *RIPK3* expression between samples with aberrant methylation (hyper- or hypo-methylated) and samples with unchanged methylation is also shown. (P) Venn diagram indicating the number of samples with aberrant methylation in each cluster, indicating a low number of overlapping samples between clusters. (B-O) Pearson correlation was used, *P* values were adjusted for multiple testing using FDR. (C-O) The Mann-Whitney test was used to compute the differences between groups.

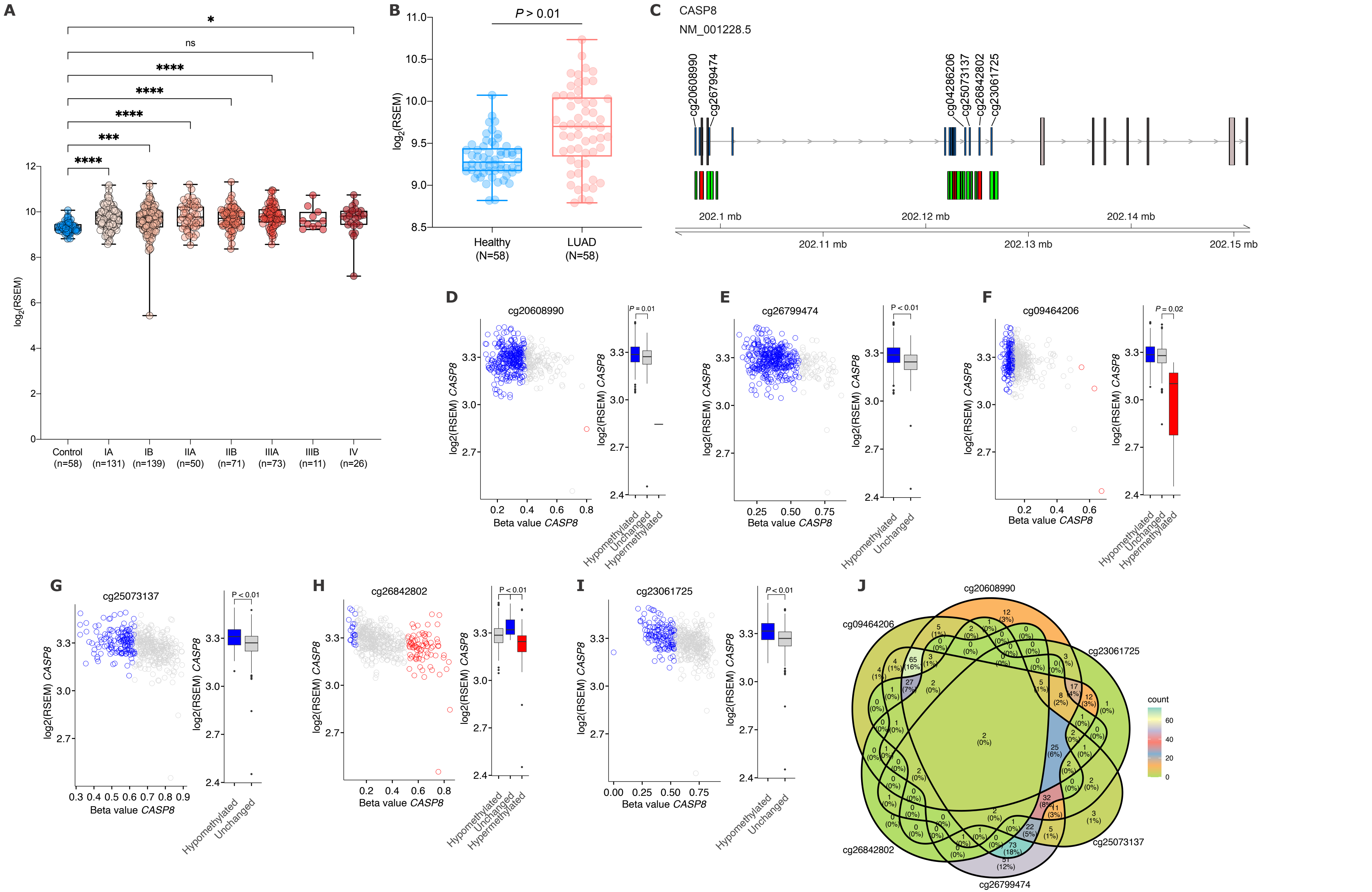

Figure S3: *CASP8* Expression and DNA Methylation Patterns in LUAD. (A) *CASP8* mRNA levels in TCGA-LUAD samples and paired healthy tissue. (B) *CASP8* mRNA levels in TCGA-LUAD according to individual tumor stages. (C) Schematic representation of the gene *CASP8*; grey boxes indicate exons; grey lines indicate introns. CpG sites along the gene are marked as blue rectangles, enhancers as green, and promoters as red rectangles below the gene schema. Labeled CpG sites show aberrant methylation patterns associated with significantly increased expression of *CASP8*. (D-I) Correlation between beta values and expression of *CASP8* for the six labeled CpG sites is shown, together with the difference in *CASP8* expression between samples with aberrant methylation (hyper- or hypo-methylated) and samples with unchanged methylation. (J) Venn diagram indicating the number of samples with aberrant methylation in each CpG site. (D-I) Pearson correlation was used; *P* values were adjusted for multiple FDR tests. (D-I) The Mann-Whitney test was used to compute the differences between groups.

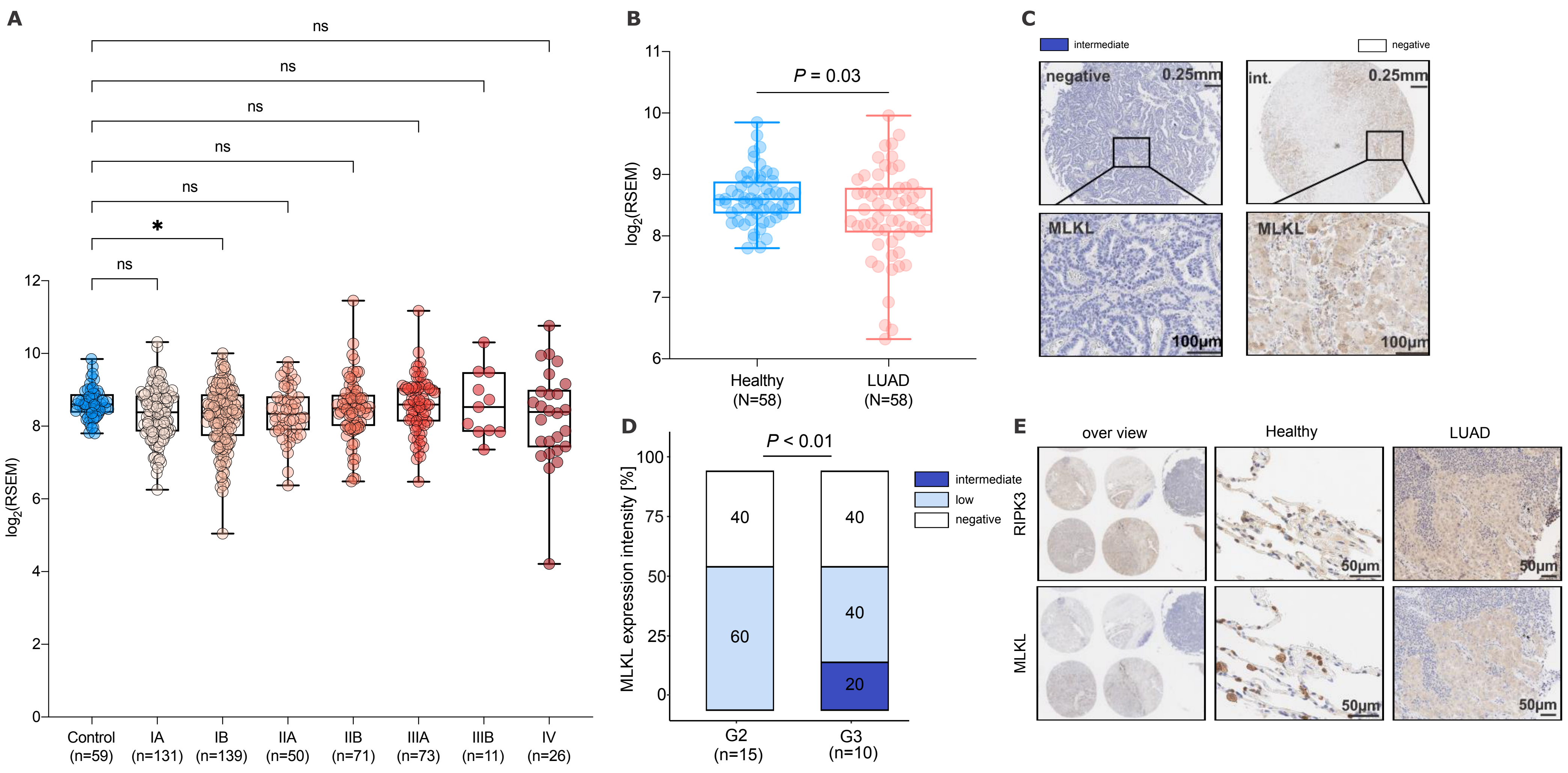

Figure S4: Alterations in MLKL in LUAD patients. (A) *MLKL* mRNA levels in TCGA-LUAD and paired healthy tissue. (B) *MLKL* mRNA levels in TCGA-LUAD according to individual tumor stages. (C) Representative tissue microarray images of MLKL staining (intermediate and negative shown). (D) Quantification of MLKL staining in 25 samples of LUAD. MLKL staining (brown) intensity was assessed and classified as weak, intermediate or absent. (E) Representative tissue microarray images showing the overview of MLKL and RIPK3 staining in one region. (A) Wilcoxon signed-rank test (B) Wilcoxon rank-sum test (D) chi-squared test.

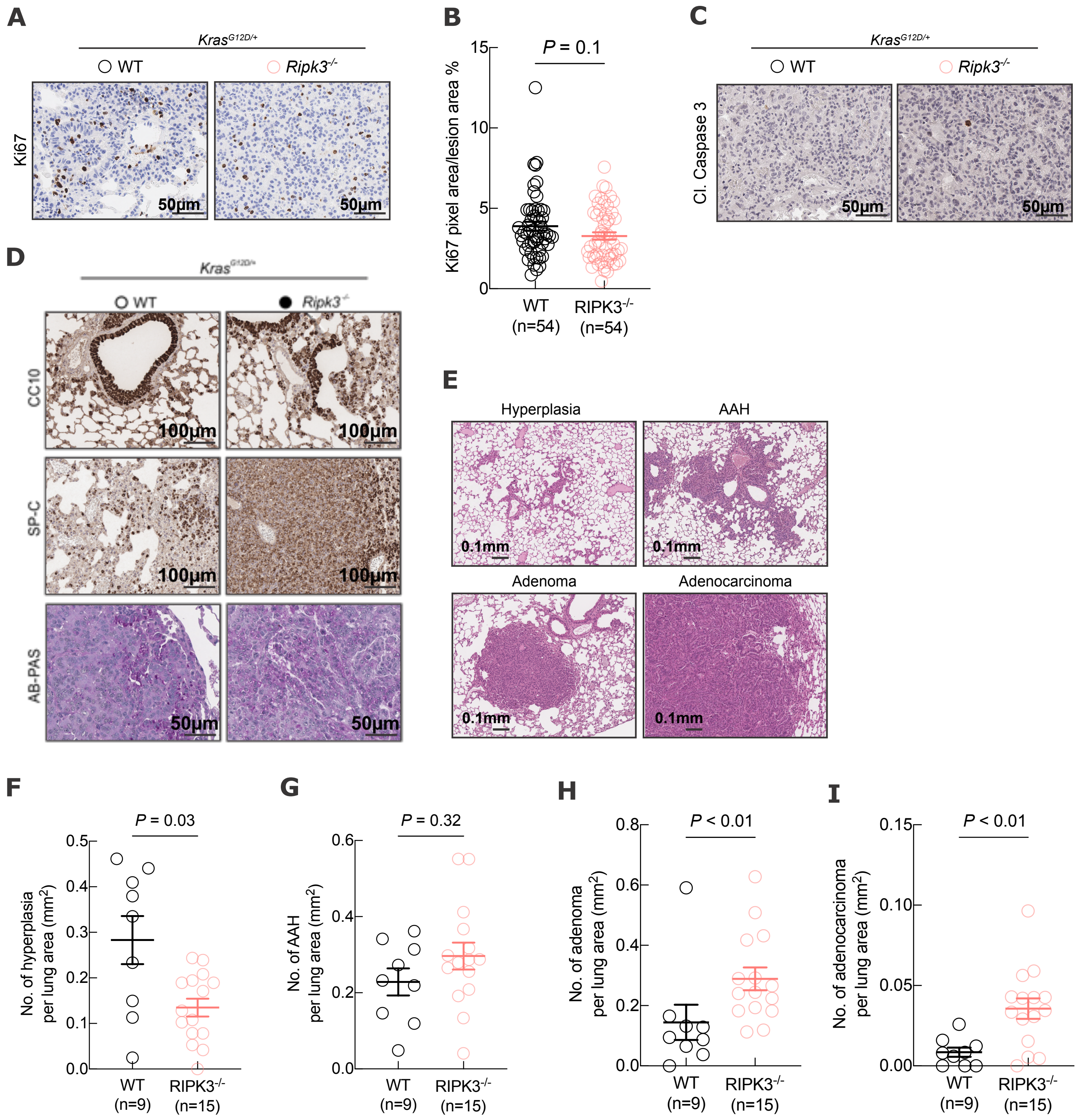

Figure S5: Ripk3 deletion exacerbates tumor burden in vivo. (A-B) Representative images (A) and quantification (B) of Ki67 positive signal in lesions from *Kras*$^{G12D/+}$ *Ripk3*$^{+/+}$ and *Kras*$^{G12D/+}$ *Ripk3*$^{-/-}$ animals. Six lesions per section per mouse were analyzed. Quantifications are represented as percentages of positive cells relative to all cells in the assessed lesions at 19 weeks post-infection with AdCRE virus. N = number of lesions. (C) Cleaved (i.e., activated) caspase-3 staining representative image of *Kras*$^{G12D/+}$ *Ripk3*$^{+/+}$ and *Kras*$^{G12D/+}$ *Ripk3*$^{-/-}$ lesions. Scale bars as reported in the figure. (D) Representative images of CC10, SP-C, AB-PAS staining of the *Kras*$^{G12D/+}$ *Ripk3*$^{+/+}$ and *Kras*$^{G12D/+}$ *Ripk3*$^{-/-}$ lesions at 19 weeks post-infection with AdCRE virus. Scale bars as reported in the figure. (E) Representative images of different grades of lesions in experimental mice. Scale bars as reported in the figure. (F-I) Quantification of the histologically graded tumor lesions, i.e., number of (F) hyperplasia, (G) atypical adenomatous hyperplasia, (H) adenomas, and (I) adenocarcinomas per lung area per mm^2 from mice of the indicated genotypes at 19 weeks post-infection. One section per animal was assessed for number of lesions for each grade. (F-I) Mann-Whitney test.

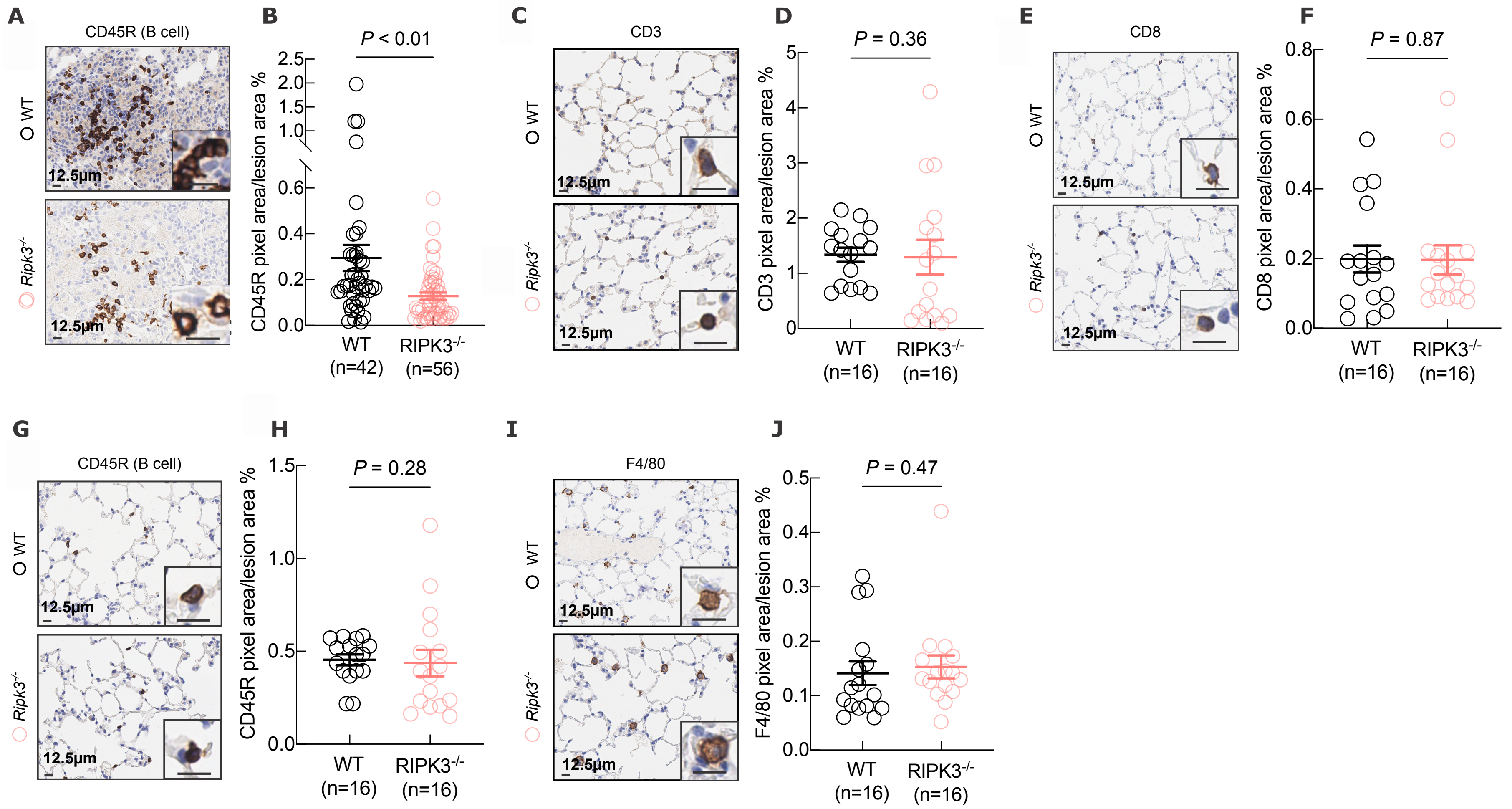

Figure S6: Ripk3 deletion alters the tumor immune microenvironment. (A-B) Representative images (A) and quantification (B) of CD45R for B cells positive signal in lesions from *Kras*$^{G12D/+}$ *Ripk3*$^{+/+}$ and *Kras*$^{G12D/+}$ *Ripk3*$^{-/-}$ animals. Six lesions per mouse were quantified. A number of individual mice is shown in the figure. Inserts are higher magnifications of the corresponding images. Scale bars as reported in the figure. (C-J) Healthy/normal tissue analyses: Representative images (C) and quantification (D) of CD3 for CD3+ T cells, (E-F) Representative images (E) and quantification (F) of CD8 for CD8+ T cells, (G-H) Representative images (G) and quantification (H) of CD45R for B cells, (I-J) Representative images (I) and quantification (J) of F4/80. Quantification of positive pixel/selected area [%], four regions of 1.2 mm2 quantified. Inserts are higher magnifications of the corresponding images. Scale bars as reported in the figure. (B, D, F, H, J) Analyzed by the Mann-Whitney test.

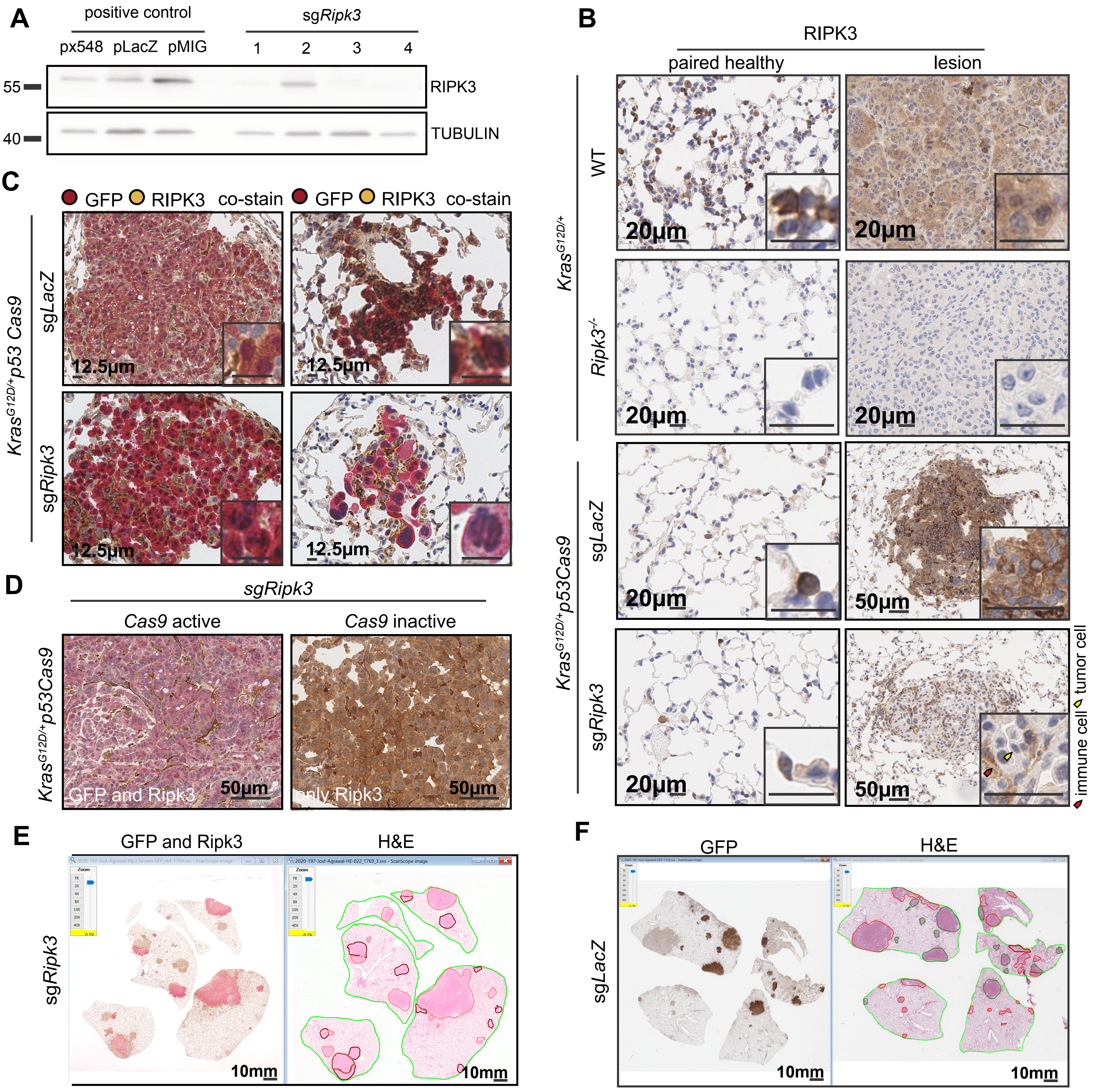

Figure S7: Ripk3 tumor-specific deletion exacerbates tumor burden. (A) Western blot of RIPK3 and α-Tubulin of four sgRNAs against *Ripk3* and positive control. (B) Representative images of RIPK3 staining in paired healthy and tumor lesions of the *Kras*$^{G12D/+}$*Ripk3*$^{+/+}$ and *Kras*$^{G12D/+}$*Ripk3*$^{-/-}$ animals at 19 weeks post-infection with AdCRE virus (upper four panels) and *Isl-Kras*^{G12D}; *p53*$^{\Delta/\Delta;}$ *Cas9* animals at 16 weeks post-infection with AAV9 virus (lower four panels). Inserts are higher magnifications of the corresponding images. Scale bars as reported in the figure. (C) Representative images of GFP and RIPK3 staining of lung from the experimental *Kras*^{G12D}; *p53*$^{\Delta/\Delta}$; *Cas9* animals. Inserts are higher magnifications of the corresponding images. Scale bars as reported in the figure, (D) Representative images of co-stain GFP, and RIPK3 staining of *Kras*^{G12D}; *p53*$^{\Delta/\Delta}$; *Cas9* animals injected with AAV9 specific to sg*Ripk3*. Scale bars as reported in the figure. (E-F) Representative images of the analysis scheme for tumor burden quantification of *Kras*^{G12D}; *p53*$^{\Delta/\Delta}$; *Cas9* animals injected with AAV9 specific to (E) sg*Ripk3* and (F) sg*LacZ*. Scale bars as reported in the figure.

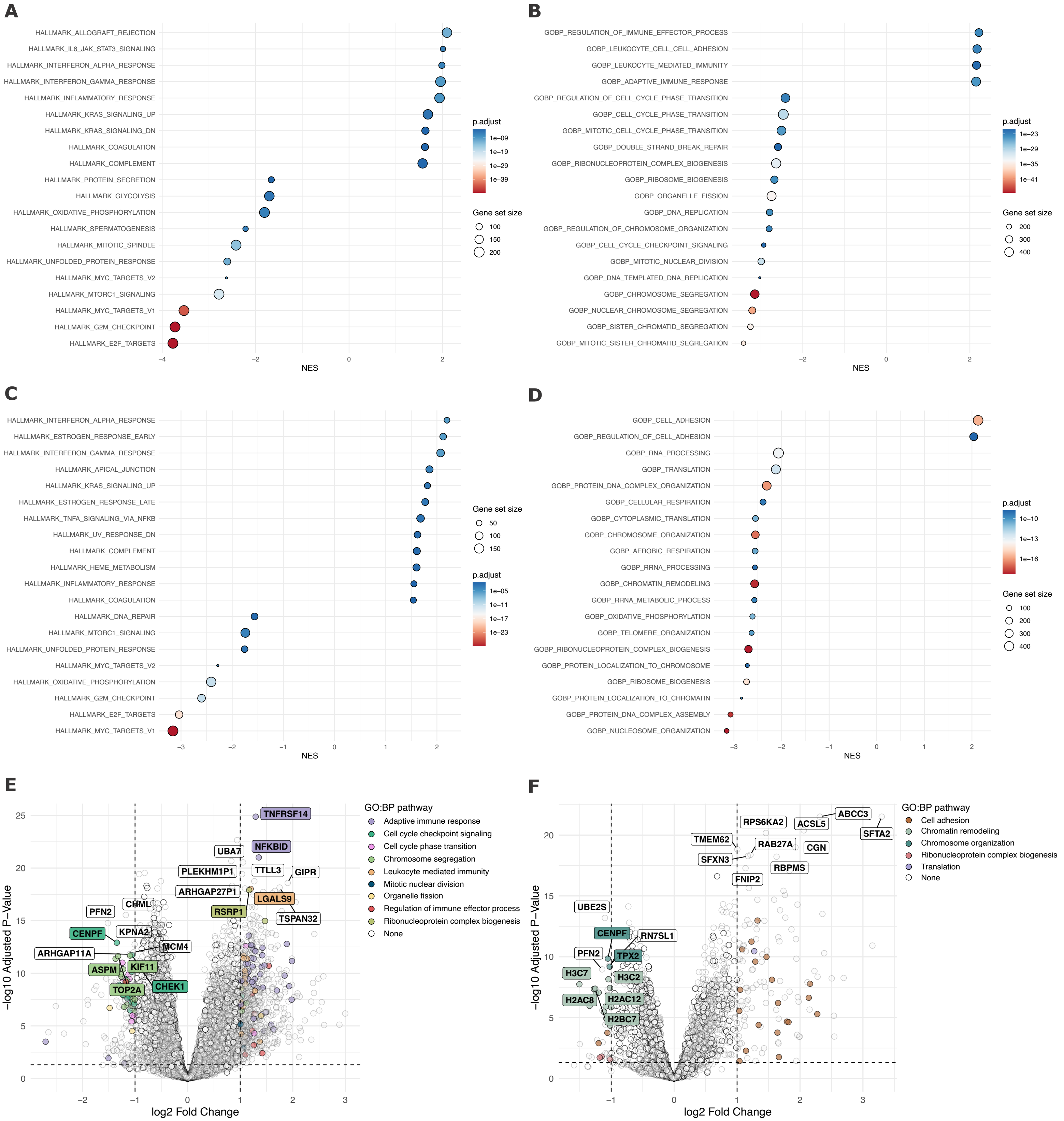

Figure S8: *RIPK3*-associated gene expression and pathway signatures across TCGA and TRACERx LUAD tumors. (A) Gene Set Enrichment Analysis (GSEA) of Hallmark gene sets in TCGA LUAD tumors comparing *RIPK3*^{hi} vs *RIPK3*^{lo} samples. Dot plot displays significantly enriched gene sets (FDR < 0.05). Normalized Enrichment Score (NES) is shown on the x-axis; dot color represents adjusted p-value, and dot size reflects gene set size. (B) GSEA of Gene Ontology Biological Process (GO:BP) terms in TCGA using the same comparison and visual scheme as in (A). (C) GSEA of Hallmark gene sets in TRACERx LUAD tumors (RIPK3hi vs RIPK3lo, visualized as in (A). (D) GSEA of GO:BP terms in TRACERx, as in (B). (E) Volcano plot of differentially expressed genes in TCGA, colored by their most strongly associated GO:BP gene set (based on highest NES). Only one gene set is assigned per gene. (F) Same as (E), but for TRACERx LUAD tumors.

Supplementary Table 1: SNVs and CNVs in TCGA-LUAD samples

SNVs

Sample	Chromosome	Start	End	Ref	Alt	Gene	Effect	Amino_Acid_Change	DNA_VAF	SIFT	PolyPhen
TCGA-05-5420-01	14	24808294	24808294	T	A	RIPK3	Missense_Mutation	p.D133V	0.09	deleterious(0)	benign(0.004)
TCGA-55-A490-01	14	24809080	24809080	G	A	RIPK3	5'UTR		0.55		
TCGA-62-8399-01	14	24805493	24805493	G	A	RIPK3	Missense_Mutation	p.S482L	0.28	tolerated(0.67)	benign(0.001)
TCGA-86-7955-01	14	24807659	24807659	G	A	RIPK3	Missense_Mutation	p.R196W	0.4	tolerated(0.07)	benign(0.002)
TCGA-95-7567-01	14	24807128	24807128	T	C	RIPK3	Silent	p.L261L	0.07		
TCGA-NJ-A4YQ-01	14	24808683	24808683	C	A	RIPK3	Silent	p.V47V	0.09		

CNVs

Sample	GISTIC Score
TCGA-44-8119-01	-2
TCGA-35-5375-01	2
TCGA-44-7667-01	2
TCGA-44-7670-01	2
TCGA-49-4487-01	2
TCGA-50-8457-01	2
TCGA-55-7910-01	2
TCGA-64-1677-01	2
TCGA-64-5815-01	2
TCGA-67-3773-01	2
TCGA-78-8640-01	2
TCGA-91-6847-01	2
TCGA-95-A4VP-01	2
TCGA-MP-A4T6-01	2

Supplementary Table S2: sgRNA sequence list

ID	Gene	Target	Target Sequence	Reverse Complement	Forward oligo seq	Reverse oligo seq
1	Ripk3	_1	GAGTTAATGATT CATTGCTG	CAGCAATGAATC ATTAACTC	caccgGAGTTAATGAT TCATTGCTG	aaacCAGCAATGAATC ATTAACTCc
2	Ripk3	_2	GGCCTGTCCACG TTTCAGGG	CCCTGAAACGTG GACAGGCC	caccgGGCCTGTCCA CGTTTCAGGG	aaacCCCTGAAACGT GGACAGGCCc
3	Ripk3	_3	GTGTAGGAAGAA GATATCCT	AGGATATCTTCTT CCTACAC	caccgGTGTAGGAAG AAGATATCCT	aaacAGGATATCTTCT TCCTACACc
4	Ripk3	_4	CGGACACGAAGT CCCACTGG	CCAGTGGGACTT CGTGTCCG	caccgCGGACACGAA GTCCCACTGG	aaacCCAGTGGGACT TCGTGTCCGc
5	lacZ	_1	GGCGTTTTCGCC GATTTGCG	CGCAAATCGGCG AAAACGCC	caccgGGCGTTTTCGC CGATTTGCG	aaacCGCAAATCGGC GAAAACGCCc
6	lacZ	_2	TCGTAGTTATCTA CACGACG	CGTCGTGTAGAT AACTACGA	caccgTCGTAGTTATC TACACGACG	aaacCGTCGTGTAGA TAACTACGAc
7	lacZ	_3	TAGATAACTACG ATACGGGA	TCCCGTATCGTA GTTATCTA	caccgTAGATAACTAC GATACGGGA	aaacTCCCGTATCGTA GTTATCTAc
8	lacZ	_4	TCATCTGTCAAC GCCGCGCC	GGCGCGGCGTTG ACAGATGA	caccgTCATCTGTCAA CGCCGCGCC	aaacGGCGCGGCGTT GACAGATGAc
